# Revisiting the Immune Balance Theory: A Neurological Insight Into the Epidemic of COVID-19 and Its Alike

**DOI:** 10.3389/fneur.2020.566680

**Published:** 2020-10-15

**Authors:** Junjiao Wu, Yu Tang

**Affiliations:** ^1^National Clinical Research Center for Geriatric Disorders, Department of Geriatrics, Xiangya Hospital, Central South University, Changsha, China; ^2^Department of Rheumatology and Immunology, Xiangya Hospital, Central South University, Changsha, China; ^3^Provincial Clinical Research Center for Rheumatic and Immunologic Diseases, Xiangya Hospital, Central South University, Changsha, China

**Keywords:** COVID-19, SARS-CoV-2, neurological complications, neuroinvasion, cytokine storm, immune imbalance

## Abstract

As the pandemic of COVID-19 is raging around the world, the mysteriousness of severe acute respiratory syndrome-coronavirus 2 (SARS-CoV-2) coronavirus is being revealed by the concerted endeavors of scientists. Although fever and pneumonia are typical symptoms, COVID-19 patients exhibit multiple neurological complications. In this interim review, we will summarize the neurological manifestations and their potential causes in COVID-19. Similar to the other two fatal respiratory coronaviruses, SARS-CoV and Middle East respiratory syndrome coronavirus (MERS-CoV), SARS-CoV-2 also shows to be neuroinvasive that may spread from the periphery to brain, probably by the retrograde axonal transport. The invaded viruses may directly disrupt the complex neural circuits, and raise a chronic activation of immune responses. In another hand, multiple organ failure in severe COVID-19 is caused by the systemic acute immune responses, and unsurprisingly caused the brain inflammation and led to encephalitis. However, in the central nervous system (CNS), the activation of resident immune cells including microglia and astrocytes may lead to chronic immune imbalance, which underlies the potential long-term effects in synaptic changes and neuropsychiatric impairments. The neuroinvasive biology also provides a possible link with the Braak staging of neurodegenerative diseases such as Parkinson's disease (PD). Although with considerable advances, the neurotropic potential and chronic neurological effects caused by SARS-CoV-2 infections merit further investigations.

## Introduction

The ongoing spread of COVID-19 disease, is the first pandemic ever caused by coronaviruses in the human history, as announced by the World Health Organization (WHO) in March 2020. The ferocious virus, isolated as a new strain of zoonotic coronavirus named as severe acute respiratory syndrome-coronavirus 2 (SARS-CoV-2), has rapidly spread with over 23.2 million confirmed cases and 0.8 million deaths globally as of Aug 23 2020 (Johns Hopkins University). The pandemic has exhausted the entire worlds' personal protective equipment and medical ventilators, and is also strenuously hurting the global economy and raising considerable social issues. As we are in the midst of this ongoing pandemic, it has gathered the concerted efforts of clinicians, public health experts, virologists, immunologists, and other scientists to understand the virus's biology and blocking agents. So far, a myriad of urgent endeavors has been maneuvered, aiming to reveal the multiple aspects of this wily virus, ranging from the genomic structures, sensing receptors to the development of specific medicines and vaccines.

Structurally, SARS-CoV-2 is a single-stranded RNA virus, whose genome contains 29,891 nucleotides in size and 12 putative functional open reading frames (ORFs) (1). Of those translated proteins, the spike proteins located on the virus surface mediate the virus entry into host cells (2, 3). Mechanistically, the spike of SARS-CoV-2 senses the angiotensin converting enzyme 2 (ACE2) receptor (2–5), the same as SARS-CoV, which normally helps regulate blood pressure and anti-atherosclerosis (6). This binding, in concert with host proteases, principally TMPRSS2, facilitates the virus getting through the cell membrane by endocytosis (4), followed by hijacking the host cell's translation machinery and producing massive virus copies and further invading new cells. As ACE2 is typically enriched in type II alveoli cells, the lung tissue becomes the major organ affected by the virus (7). The typical symptoms of COVID-19, unsurprisingly, are fever, cough, and pneumonia, which probably lead to acute respiratory distress syndrome (ARDS) and acute lung injury, as described in around 20% of COVID-19 patients (8).

Along with SARS-CoV broke out in 2003 and Middle East respiratory syndrome coronavirus (MERS-CoV) since 2012, SARS-CoV-2 is the third coronavirus that can cause severe respiratory diseases. Genomic analysis shows that SARS-CoV-2 is in the same β-coronavirus clade as SARS-CoV and MERS-CoV, and shares a highly homologous sequence with SARS-CoV (9). Scientists thus have put great efforts in clarifying how it resembles and differs from SARS-CoV and MERS-CoV at multiple levels that may shed light on the COVID-19 therapeutics and drug repurposing. Specifically, the similarity goes to the systemic organ injury and cytokine storm in severe situations.

## Systemic Organ Injury

Although the symptoms in lungs are manifested at an early stage, they can be extended to multiple organs including the blood vessels, heart, gut, kidneys, testicles, and brain, which are well-known to express ACE2 and are potential targets of COVID-19 (10). Unlike the outbreak of SARS and MERS, the emerging single-cell RNA-sequencing (scRNA-seq) during recent years is rapidly advancing our ability to comprehensively map the cell types with ACE2/TMPRSS2 expression (11–15). It is shown that, besides pneumocytes, ACE2 receptors are present in various cell types including the nasal epithelial cells, oral mucosa epithelial cells, cholangiocytes, intestine enterocytes and, importantly, immune cells such as B cells, Natural killer T cells, monocytes, and macrophages (11, 13, 16–19). Notably, ACE2 is also expressing in the brain, in which eight cell types were identified including excitatory neurons, inhibitory neurons, oligodendrocyte progenitors, oligodendrocytes, microglia, astrocytes, pericytes, and endothelial cells (13). However, other studies showed contradictory results that glial cells may not express ACE2, but instead might express non-canonical docking receptors such as Basigin (BSG) or Neuropilin-1 (NRP1) to facilitate viral cell entry and replication (20, 21). Nevertheless, the present of ACE2 receptors in multiple organs underlies the systemic impairment by SARS-CoV-2 infection.

## Neurological Manifestations

Coronavirus infection has been associated with neurological manifestations such as stroke, seizure, convulsions, mental confusion, and encephalitis (22, 23). During the outbreak in 2003, SARS-CoV could induce neurological diseases such as polyneuropathy, encephalitis, and aortic ischemic stroke (24). The virus itself has been detected in the cerebrospinal fluid (CSF) samples, and even the brain of deceased patients (25, 26). In 2012, nearly 20% of patients with MERS-CoV infection developed neurological symptoms, including ischemic stroke, paralysis, unconsciousness, Guillain-Barre syndrome, and other infectious neuropathy (27). It is thus not surprising to see neurological manifestations in COVID-19 patients as well (28–30). In general, COVID-19 neurological manifestations could be classified into two categories: central nervous system (CNS) symptoms and peripheral nervous system (PNS) symptoms. CNS symptoms included headache, dizziness, acute cerebrovascular disease, ataxia, disturbance of consciousness, and epilepsy. However, PNS symptoms are less severe and manifested as neuralgia, hypoplasia, hyposmia, and hypogeusia. Notably, respiratory illness in COVID-19 patients may also result from the direct role of SARS-CoV-2 in respiratory control nuclei in the brain (31). Interestingly, still many patients who present with severe neurological complications have hardly any respiratory symptoms, suggesting a rather heterogenous neurological responds among individuals, and that neurological manifestations did not appear concomitantly with respiratory symptoms.

In a retrospective series of 214 COVID-19 patients at a hospital located in the epicenter of Wuhan, China, neurologic symptoms were recorded in 78 patients (36.4%) included headache and disturbed consciousness, and 6 patients had strokes (32). Half of the patients in Strasbourg, France by severe SARS-CoV-2 infection was associated with encephalopathy, prominent agitation and confusion, and some of them had single acute ischemic strokes after brain imaging (33). In Japan, a COVID-19 patient was brought in by the ambulance due to a convulsion accompanied by unconsciousness, which was diagnosed with aseptic meningitis/encephalitis (34). Notably for this case, the specific SARS-CoV-2 RNA was detected from the CSF sample. Similarly, a medical team at a hospital in Beijing, China confirmed the presence of SARS-CoV-2 in the CSF of a 56-year-old patient with COVID-19 by genome sequencing, thereby clinically verifying viral encephalitis (35). Notably, unlike encephalopathy, the acute stroke is most likely caused by endothelial injury due to a pro-inflammatory hypercoagulable state post SARS-CoV-2 infection (36, 37). Hence in China, the neurological symptoms have been added into the *Diagnosis and Treatment Protocol for 2019 Novel Coronavirus Pneumonia* (The 7th Trial Edition), released by National Health Commission & State Administration on March 3, 2020, which reminds us of taking nucleic or genomic tests with CSF samples and carefully handling with neurological complications to reduce the fatality of critical care patients.

**Figure 1:**
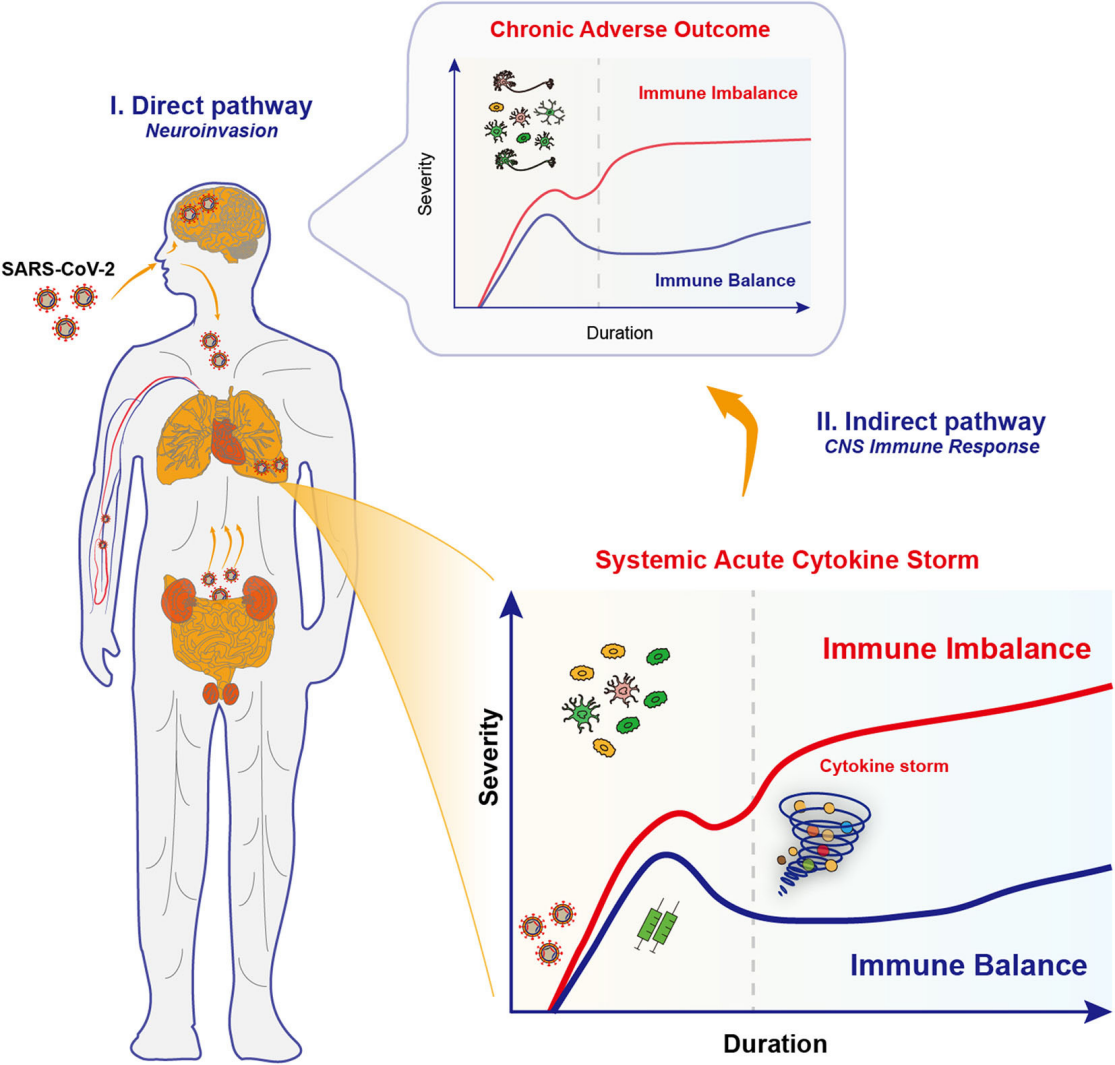
Proposed neuroinvasion routes and immune responses in COVID-19. Upon infections by SARS-CoV-2 coronaviruses, COVID-19 patients exhibit multiple neurological complications, which might be due to the effects through the direct pathway and the indirect pathway. **(I)** The neuroinvasive properties of SARS-CoV-2 underlies the retrograde axonal transport in the direct pathway. Specifically, SARS-CoV-2 viruses may go upward through the olfactory nerve across the cribiform plate and to the brain, or alternatively, start from the gastrointestinal system to invade the enteric nervous system and finally the brain. Several other invasion routes for SARS-CoV-2 may include blood-borne diffusion through the blood-brain barrier, blood-cerebrospinal fluid barrier and meningeal cerebrospinal fluid barrier. Those invaded viruses may directly disrupt the complex neural circuits, and raise a chronic activation of immune responses. **(II)** Multiple organ failure in severe COVID-19 is caused by the systemic acute immune responses, the cytokine storm, and unsurprisingly caused the brain inflammation and led to encephalitis. However, the potential long-term effects in synaptic changes and neuropsychiatric impairments in key brain regions should not be neglected. This is probably caused by the activation of CNS immune cells that renders chronic immune imbalance.

Those neurological manifestations observed in COVID-19 patients are reminiscent of neuroinvasive potential of SARS-CoV-2 virus, like the other zoonotic coronaviruses SARS-CoV and MERS-CoV (31). An increasing number of patients with COVID-19 reported a sudden loss of smell (anosmia) or taste (dysgeusia) (38, 39) that may serve as initial manifestations and warning signs for possible subsequent CNS involvement. Given that ACE2 is highly expressed in nasal epithelial cells (11), people speculate that nose might be the first stop during the invasion of viruses, which then go upward through the olfactory nerve across the cribiform plate, and to the brain (29) (Figure 1). One recent study showed that, based on bulk and single-cell RNA sequencing, ACE2 expressed in supporting and stem cells in the human/mouse olfactory epithelium, as well as vascular pericytes in the mouse olfactory bulb, however, was not detected in olfactory sensory or bulb neurons (40). Furthermore, autopsy studies of COVID-19 patients found that olfactory epithelium showed prominent leukocytic infiltrates in the lamina propria and focal atrophy of the mucosa, and olfactory nerve fibers in the lamina propria were lack of myelin, suggestive of axonal damage (41). However, the clear evidence is still lacking to confirm whether the olfactory neuropathy is due to direct viral infection or mediated by perturbing supporting non-neural cells.

The occurrence of diarrhea, as another COVID-19 symptom, suggests that the gastrointestinal system is a possible alternative pathway to invade the enteric nervous system and finally the brain (42) (Figure 1). Several invasion routes for coronaviruses have been postulated (28, 43), including retrograde axonal transport, blood-borne diffusion through the blood-brain barrier (BBB), blood-cerebrospinal fluid barrier, and meningeal cerebrospinal fluid barrier (44). Once in the brain, those viruses may directly destroy the complex organization of neural circuits through neuronal damage, and raise a chronic activation of the inflammatory responses.

## Cytokine Storm

Apart from the direct infection of the brain, SARS-CoV-2 may cause neurological disorders indirectly by triggering an over-activated immune responses, characterized as cytokine storm. Cytokines are chemical signaling molecules that summon immune cells and mediate a balanced immune response, however, in the cytokine storm, levels of certain cytokines soared far beyond the required levels so that the recruited immune cells began to attack healthy tissues and caused catastrophic organ failures. Vital research suggests that for many patients who died from COVID-19, the fatal blow may be their own immune system rather than the virus itself.

The initiation of cytokine storm is a common complication caused by fatal respiratory coronaviruses, like SARS-CoV and MERS-CoV, and is the major cause of morbidity (45). Studies have shown that increased numbers of pro-inflammatory cytokines (such as IL-1β, IL-6, IL-12, IFN-γ, IP10, and MCP1) in the serum of severe SARS patients are associated with lung inflammation and extensive lung injury (46). Similarly in 2012, it was reported that MERS-CoV infection can induce substantially elevated concentrations of pro-inflammatory cytokines such as IL-6, IFN-γ, TNF-α, IL-15, and IL-17 (47, 48). Ongoing studies have also been revealing the features of cytokine storms in COVID-19 patients. For most severe patients with COVID-19, the levels of pro-inflammatory cytokines soared in the serum, similar to that in SARS and MERS, including IL-6, IL-1β, IL-2, IL-8, IL-17, G-CSF, GM-CSF, IP10, MCP1, MIP1α, and TNF-α (8, 49–53). In addition, patients admitted to the intensive care unit (ICU) had higher G-CSF, IP10, MCP1, MIP1A, and TNF-α concentrations than patients not admitted to the ICU, suggesting that cytokine storm is associated with disease severity (8).

High levels of pro-inflammatory cytokines could cause shock and tissue damage, leading to respiratory failure, or multiple organ failure. They mediate extensive lung pathology, resulting in massive infiltration of neutrophils and macrophages, diffuse alveolar injury and the formation of clear membranes and diffuse thickening of the alveolar wall (54). Thus, it is urgently needed therapeutics based on suppressing cytokine storms. In the clinical practice, anti-inflammatory agents have been frequently used for the treatment of patients with severe illness by virus infections. For instance, corticosteroids were ever used in treating patients with SARS, which have actually saved many lives and families. However, long-term use of this powerful broad immunosuppressant can cause various complications such as increased cholesterol, brittle bones, cataracts, as well as depression that may greatly reduce the quality of life. More unfortunately, the latest evidence from SARS and MERS patients shows that receiving corticosteroids has no effect on mortality, but delays viral clearance (8, 55, 56). Therefore, according to the WHO's interim guidelines, corticosteroids should not be routinely given systemically.

It is noteworthy that, IL-6, one of the cytokines elevated in response to SARS-CoV-2 was the most reported in multiple clinical groups. For instance, in a series of 99 COVID-19 cases from hospitals in Wuhan and Shanghai, China, half of the patients show elevated IL-6 levels (57). Another group investigated the immune responses and cytokine release from patients in Chongqing, China. They found that IL-6 was higher in 76.19% of severe patients, whereas that was seen in only 30.39% of mild patients (58). It echoes that the elevated serum IL-6 correlates with pneumonia, ARDS, and adverse clinical outcomes (59–61). Elevated serum C-reactive protein, which is regulated by IL-6, also serves as a biomarker of severe coronavirus infection (62). Based on this fact, drugs such as Tocilizumab, Satralizumab, and Sarilumab, as IL-6 receptor (IL-6R)-targeted monoclonal antibodies (mAbs), might prove beneficial for the treatment of COVID-19 (63). Indeed, controlled clinical trials are underway around the world to test the treatment of IL-6 and IL-6R antagonists for COVID-19 patients with severe respiratory complications. Preliminary results from the study of 21 severe COVID-19 patients receiving Tocilizumab in Anhui province, China are encouraging (64). All patients have recovered from fever within the first day of Tocilizumab treatment (64). Other clinical trials are also underway in different countries. Although the efficacy of Tocilizumab in COVID-19 patients with ARDS requires further evaluation in a larger randomized controlled trial, this encouraging clinical trial suggests that neutralizing mAbs against other pro-inflammatory cytokines such as IL-1 and IL-17 might also be useful (54). For urgently treating the soaring number of severe patients, the *Chinese Diagnosis and Treatment Protocol for 2019 Novel Coronavirus Pneumonia* (The 7th Trial Edition) has updated a guideline for taking immunotherapy: for patients with extensive lung lesions and severe cases who also show an increased level of IL-6 in laboratory testing, Tocilizumab can be used for treatment. Although with exciting benefits, the inhibition of IL-6 pathway works mostly for severe cases, the long-term treatment strategy against the SARS-CoV-2 infection requires the rapid development of effective anti-viral drugs and, more importantly, vaccines.

## CNS Immune Responses

The systemic cytokine storm caused the multiple organ failure, and unsurprisingly triggered the hyperinflammatory responses inside the CNS that further exacerbated the neurological pathology. The spreading of infected leukocytes might across the compromised BBB, caused by increased pro-inflammatory cytokines, from the periphery to the brain. Previously, for most cases of SARS, autopsy detections of affected brain tissue samples have shown signs of extensive edema, microglial hyperplasia, neuron necrosis, nerve demyelination, as well as massive infiltration of monocytes and lymphocytes (65). Based on recent autopsy results, brain hyperemia and edema, partial neuron degeneration, as well as inflammatory cell infiltration in perivascular regions were also detected in COVID-19 patients from China (66). The persistence of coronavirus infection and its ability to infect macrophages, microglia, and astrocytes in the CNS are particularly critical in the pathogenesis of encephalitis (30). Notably, a neurotropic virus could directly activate glial cells and induce a pro-inflammatory phenotype (67). Studies have confirmed that primary glial cells cultured *in vitro* released a large number of pro-inflammatory factors, such as IL-6, IL-12, IL-15, and TNF-α, upon coronavirus infection (22).

Glial cells, as resident immune cells of the CNS, normally take a role in maintaining the homeostasis, responding promptly to CNS injuries such as trauma, ischemia, and infection, and also providing support and protection for neurons. Particularly, microglia are initially activated to clear the invaded pathogens by secreting pro-inflammatory mediators, followed by promoting tissue reconstitution and inflammation resolution. Microglia have been demonstrated to protect against lethal coronavirus encephalitis in mice (68). During the early days after infection, microglia were required to limit mouse hepatitis virus (MHV) replication and subsequent morbidity and lethality. The chemical depletion of microglia led to increased viral replication in the brain and caused ineffective T cell responses, reminiscent of the critical role of microglia in the early innate responses to virus infections (68). However, in addition to protective effects, microglia may also mediate hippocampal presynaptic membrane damage through complement system, resulting in long-term memory impairment and cognitive decline in patients with encephalitis, caused by coronavirus or human immunodeficiency virus (HIV) infection (69).

Thus, beyond the acute cytokine storm, the activation of immune cells in the brain might cause chronic inflammation and brain damages in COVID-19 patients. Taken together, in the short-term, SARS-CoV-2 infections may cause the CNS inflammation and lead to encephalitis. Potential long-term effects, such as changes in mood and cognitive behavior, and continuous changes in the expression of genes that regulate synaptic activity in key brain regions should not be neglected. Moreover, this speculation has drawing increasing attentions of clinicians and neuroscientists (21, 70–73). Hence, prognostic research on potential and longitudinal potential COVID-19-related neuropsychiatric diseases is crucial in disease surveillance and evidence-based treatment strategies (74).

## Linking With Neurodegenerative Diseases

The multiple organ failure in COVID-19 is associated with the acute immune imbalance, whereas the chronic immune imbalance in the CNS, either by invaded virus or by infiltrated immune mediators, might be happening (Figure 1). An emerging hypothesis states that the inflammation caused by viral infection may trigger and propagate chronic neuronal dysfunction, which is an event before the clinical onset of multiple neurodegenerative diseases such as Parkinson's disease (PD) and Alzheimer's disease (AD) (75). Notably, the chronic immune imbalance is the shared hallmark for neuropsychiatric and neurodegenerative diseases, due to the uncontrolled skewing of glial phenotypes into detrimental states (76). Experimental vaccination of mice with H5N1 influenza virus can mimic many aspects of PD-like initiation and pathogenesis (77, 78). The continued inflammation that follows in the viral trajectory leads to dysfunction or degeneration of dopaminergic neurons in the midbrain, just as seen in PD patients (77, 78). Therefore, it would be interesting to probe the relationship between the immune responses upon coronavirus infections and neurodegeneration/ neuropsychiatric impairments.

The similar set of sustained elevated pro-inflammatory cytokines or chemokines, typically ILs, CXCLs, and TNF, that trigger the cytokine storm of COVID-19 are also frequently detected in the CSF and autopsy brain samples (79–81), which is critical in the development and progression of numerous neurodegenerative disorders. Since the role of neuroinflammation and specific inflammatory mediators have been recently extensively reviewed in respective diseases including PD, AD, amyotrophic lateral sclerosis (ALS), Huntington's disease (HD), and multiple sclerosis (MS) (82–86), we will not discuss in much details but give some examples. For AD, pro-inflammatory factors are responsible for the increased amyloid precursor protein (APP) production and amyloid-β (Aβ) load, as well as tau hyperphosphorylation, the hallmarks of AD. Specifically, TNF-α can increase Aβ burden by promoting β-secretase production and increased γ-secretase activity (87). Elevated IL-6 levels have been shown to activate CDK5, a kinase that phosphorylate tau proteins (88). Such extensive neuroinflammation thus would cause neuronal death that leads to cognitive impairment and dementia.

Alpha-synuclein (α-synuclein), a major component of Lewy bodies in the pathogenesis of PD, plays an important role in mediating innate and adaptive immunity (89). Particularly, the mutant forms of α-synuclein in PD could induce microglial activation, releasing various pro-inflammatory cytokines (IL-6, IL-1β, and TNF-α etc.) and CXCL12, by recognizing toll-like receptors (TLRs) (90, 91). Similarly, for ALS, the aggregated proteins as mutated superoxide dismutase (mSOD1), caused motoneuron injury and triggered microgliosis in spinal cord cultures by releasing pro-inflammatory cytokines and free radicals (92). Overall, the aggregated proteins among multiple neurodegenerative diseases including α-synuclein, Aβ, and mSOD1, can initiate a pro-inflammatory responses that lead to a sustained imbalance of neuroinflammation and neuronal loss due to the persistent protein aggregations (76).

In addition, the nerve demyelination was also observed in both SARS-CoV and SARS-CoV-2 infected patients, resembling the pathology of MS, which is also tightly associated with neuroinflammation (85). A similar pattern of elevated pro-inflammatory factors (IL-6, IL-8, TNF etc.) was recorded in the CSF samples of MS patients with severe gray matter damage (93). Interestingly, other neuropsychiatric diseases such as schizophrenia, bipolar disorders, depression, and among others, are also tightly linked with the neuroinflammatory responses (94). For instance, the levels of pro-inflammatory mediators including IL-6, IL-8, IL-1β, and TNF-α in the CSF or peripheral blood are obviously higher in schizophrenia patients (95, 96). Microglia that release pro-inflammatory factors such as TNF-α can promote the release of glutamate to induce oligodendrocyte dysfunction, resulting in abnormal neural networks in the brain of schizophrenic patients (97). Notably, the altered mental status and neuropsychiatric presentations were recorded in COVID-19 patients and other coronavirus infected diseases (98, 99).

Above all, the neuroinflammation imbalance toward pro-inflammatory states shows to be a shared hallmark of various neurological diseases, hence, the CNS infiltrated immune mediators in COVID-19 patients would probably take part in the chronic pathogenesis process and bring about certain irreversible neuronal impairments.

In another hand, given that SARS-CoV-2 viruses have invaded the CNS and can be detected in the CSF, their direct effects in the chronic modifications of neural circuits worth further investigations. Also, it is intriguing to address whether the infection increases the risk of developing chronic neurodegenerative diseases. The Braak hypothesis regarding the etiology of sporadic PD proposes that neurotropic viruses entering the nasal cavity and gastrointestinal tract may trigger Lewy pathology, which then spreads to the CNS through transneuronal transport, resulting in neurodegeneration in critical brain nuclei (100). Recent studies have confirmed the nasal-brain and gut-brain deliveries in the pathogenesis of PD (101, 102). Interestingly, the symptoms of anosmia and diarrhea of COVID-19 patients indicate the nasal and digestive system as the routes of viral infection, which may echo the Braak staging evidence that the prodromal or preclinical stage of PD is characterized by olfactory and gastrointestinal symptoms (103).

## Perspective

Even though COVID-19 respiratory tract infections and cardiovascular events are the main causes of death, the clinical awareness of neurological impairments can reduce the mortality of infected patients. To reduce the risk of those neurological complications, further investigations are needed to determine specific risk factors or protective determinants of neurological events. Although recovery from the acute phase of infection can of course be relieved from a public health perspective and help stop the spread of infection, the long-term neurological effects of the disease must also be considered. So far, mounting studies have reported various neurological manifestations, however, the neurotropic potential and chronic neurological effects of the SARS-CoV-2 virus remains to be fully addressed.

Currently, the urgent need for treating COVID-19 severe patients is still suppressing cytokine storms and balancing the immune system, particularly also in the CNS. Unfortunately, no specific medicine against COVID-19 has been developed till now. Apart from using mAbs such as Tocilizumab, Satralizumab, and Sarilumab, a recent study reported that dexamethasone, a corticosteroid used widely for its anti-inflammatory and immunosuppressant effects, showed to reduce the mortality by 1/3 among patients receiving invasive mechanical ventilation and by 1/5 among patients receiving oxygen by other means, but had no effects for patients without receiving respiratory support (104). However, this drug, as mentioned earlier as other corticosteroid drugs, was also under critical concerns of side effects. Different drugs work depending on the severity of disease and the timepoint for delivery. Adding the need of treating neurological complications, the therapeutic strategy becomes more complicated. It is possible that the anti-neuroinflammatory drugs that used for treating neurodegenerative diseases might be repurposed, due to their capability of crossing the BBB. However, the candidate drugs and doses would be really dependent on each individual, since neurological complications were heterogenous among populations, and importantly, their safety for normal people infected with SARS-CoV-2 will also await clinical trials to be proven.

Additionally, another method to alleviate the fierce immune responses is employing the anti-inflammatory and anti-apoptotic effects of mesenchymal stem cells (MSCs), which can repair lung epithelial cell damage and facilitate alveolar fluid clearance (54). So far, they are still in clinical trials and are waiting for evaluation. In the other way, fortunately, the development of vaccines for the public has been right on the track (105–108), some of which have been under Phase III Clinical Trials.

Lastly, while we are talking about the acute or chronic immune imbalance, it is better to appreciate that keeping the immune system in balance is pivotal for maintaining health from infections and other pathogenic agents. To achieve this goal, people should lead a healthy lifestyle, with diets rich in whole grains, vegetables, and fruits, but low in red meat and high-fat foods. Regular exercise and stress relief are also incorporated, so as to strengthen our immunity against viral infections.

## Author Contributions

YT conceived the manuscript. JW and YT wrote and revised the draft. All authors approved the submission.

## Conflict of Interest

The authors declare that the research was conducted in the absence of any commercial or financial relationships that could be construed as a potential conflict of interest.
